# Nasal septum: an extremely unusual location for head and neck paraganglioma^☆^

**DOI:** 10.1016/j.bjorl.2016.04.023

**Published:** 2016-06-03

**Authors:** Surendra Gawarle, Prashant Keche, Subhro Ganguly

**Affiliations:** Shri Vasantrao Naik Government Medical College, Department of Otorhinolaryngology, Yavatmal, India

## Introduction

Paragangliomas are slow growing, benign tumours arising from embryonal neural crest cells. These neuroendocrine tumours, mostly originate from the adrenal glands in 90% cases in the form of Pheochromocytoma. Only 5–10% of these tumours are extra-adrenal and they can be located anywhere between neck and pelvic region along the distribution of sympathetic nervous system.^1^ Only 3% of the extra-adrenal paragangliomas are found in head and neck region. Most prevalent site is carotid body giving rise to the classical Chemodectoma. Other sites are tympano-jugular region, vagal region, trachea, tongue, larynx, hypophyses, pineal gland and orbit.^2^ A recent review identified only 25 such cases worldwide and 12 of them were located in the nasal cavity.^3^ In the nasal cavity the literature showed only three cases taking origin from nasal septum.^4^ We discuss the clinical presentation and management protocol of a 15 year old patient diagnosed as paraganglioma of nasal septum with review of the literature.

## Case report

A 15 year old boy visited our centre with a history of left sided nasal obstruction gradually progressive over a period of two months with recurrent history of bleeding from left nostril. There was no history of bleeding from any other sites or easy bruisability. On anterior rhinoscopy a single smooth surfaced fleshy mass was seen filling the left nasal cavity reaching up to anterior end of inferior turbinate deviating the septum to the right side. The mass was firm, sensitive to touch and pain, bleeding on touch. Posterior rhinoscopy did not reveal any choanal extension. Rigid nasal endoscopy was performed and the mass was seen attached to the postero superior part of the left side of the nasal septum.

**Figure 1:**
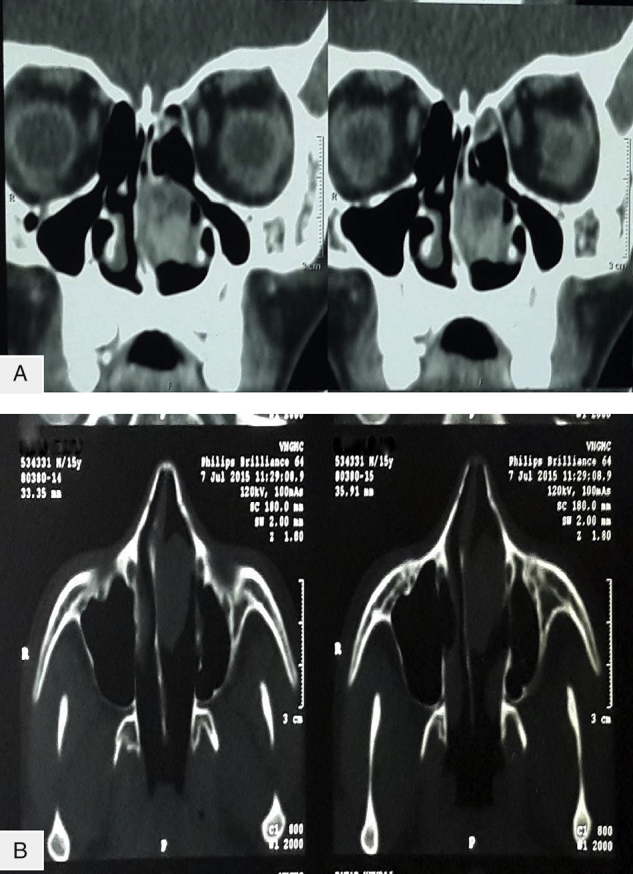
Preoperative CT scans (A) coronal and (B) axial views showing a soft tissue density lesion in left nasal cavity with heterogenous contrast enhancement.

Complete blood count, biochemistry parameters and coagulation profile were all within normal limits. Computed Tomography (CT) scan of paranasal sinuses showed a homogenous soft tissue lesion measuring 41 mm × 32 mm × 24 mm in left nasal cavity. All the sinuses were clear. No bony erosion was seen on CT scan. On contrast study, there was heterogeneous enhancement indicating a vascular tumour (Fig. 1).

**Figure 2:**
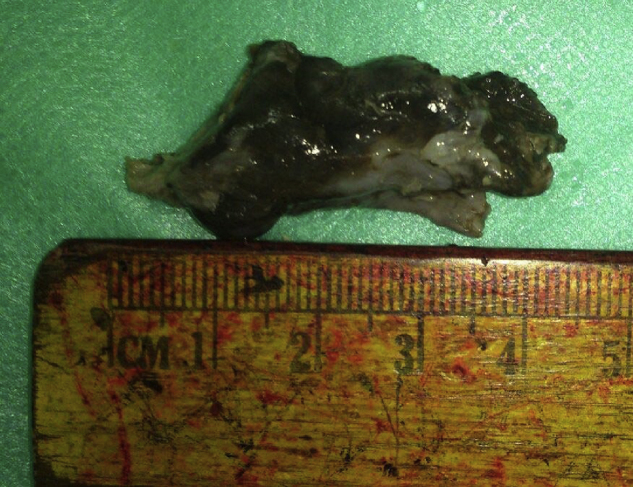
Photographs of the excised mass.

Given the vascularity of the tumour on CT scan and considering the patient's age and gender nasal angiofibroma was suspected and hence biopsy was not taken and the patient was posted for endoscopic excision of the nasal mass under general anaesthesia after taking informed consent. The mass was removed completely by a regular endoscopic approach with removal of a cuff of septal perichondrium (Fig. 2). The operative procedure and the post-operative recovery periods were uneventful.

**Figure 3:**
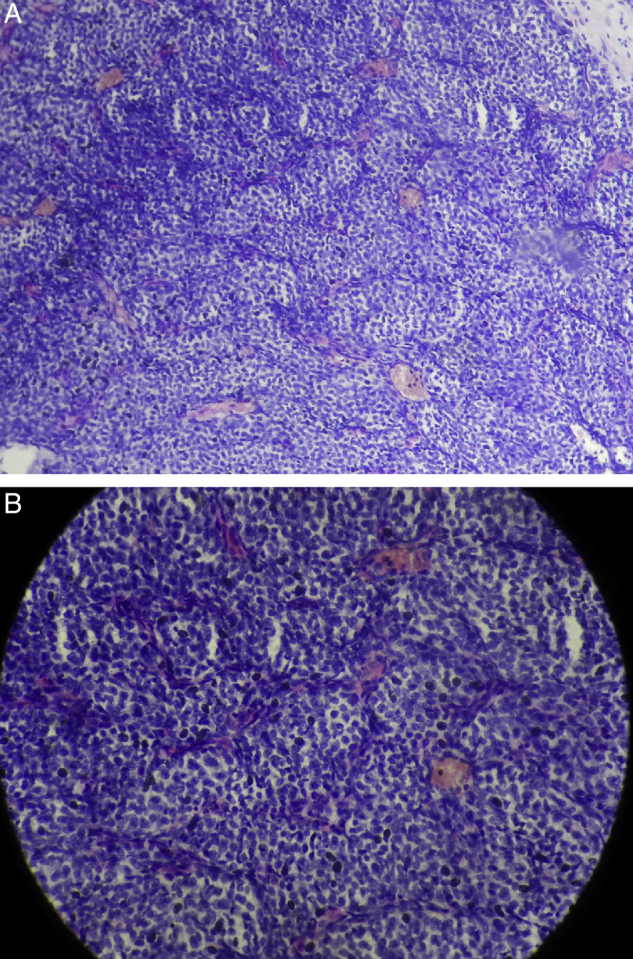
Histopathology pictures showing classical Zellballen pattern pathognomic of paraganglioma. (A) Low magnification; (B) high magnification.

Histopathology of the excised specimen showed round to oval shaped tumour cells with moderate pale cytoplasm with round vesicular nuclei with dispersed chromatin. The tumour cells were arranged in nest or classical *Zellballen* patterns which are separated by vascularised connective tissue stroma. There were no mitotic figures and cellular atypia (Fig. 3). The tumour cells showed strong and diffuse immunoreactivity for synaptophysin and focally positive for S100 protein and negative for Chromogranin, CD 10 and EMA. The diagnosis of paraganglioma was confirmed. A regular follow-up for a period of one year showed no signs of recurrence.

## Discussion

Pheochromocytoma is usually a functional tumour and patients present with symptoms of catecholamine excess but paragangliomas of head and neck region are non-functional or non-chromaffin tumour.^5^ The recognition of paragangliomas in the sino-nasal tract is chiefly based on the awareness of their occurrence in this region. Knowledge of the distribution of normal paraganglionic tissues is of paramount importance in predicting the sites of origin of paragangliomas. It is also essential to understand that paragangliomas can occur outside the domain of sympathetic and parasympathetic nervous system, probably explaining the existence of paragangliomas in unusual locations.

The origin of nasal paragangliomas is a controversial entity. Talbot et al. described paraganglionic cells at the distal maxillary artery in stillborn children supporting the theory of persistence of paraganglionic tissues along cranial arteries which could lead to development of head and neck paragangliomas.^6^ Other authors suggested that the tumour could originate from pterygoid ganglion.^3^

Nose and paranasal sinuses are the least common sites for this neoplasm in head and neck region. Nasal cavity is more commonly affected by the tumour than the sinuses. Lateral nasal wall, middle turbinate and ethmoid sinus are the commonly involved areas.^7^ Nasal septum is a very rare location for this tumour with very few cases reported in the literature previously.^4^

Nasal paragangliomas are slow growing tumours with usual interval between the onset of symptoms and final diagnosis of two years or more. The tumours tend to be bilateral and multicentric and their incidence is 3% and it goes up to 26% among patients with positive family history which corroborates with genetic predisposition.^8^

Clinically patients present with mild to profuse recurrent epistaxis, nasal obstruction, rhinorrhoea and facial oedema. The tumours have been reported in patients aged between 8 to 89 years with middle aged females being most commonly affected. Anterior rhinoscopy reveals a reddish polypoidal mass in nasal cavity extending into the sinuses. CT scan reveals a soft tissue density expansile mass seen in nasal cavity and para-nasal sinuses with heterogenous contrast enhancement. Bony erosion is seen in case of malignant paragangliomas.^9^

Macroscopically paragangliomas are firm to hard, encapsulated tumours, greyish or rosy in colour. Microscopically the tumour consists of epithelioid cells also called chief cells with round nuclei and eosinophilic cytoplasm forming a nest like pattern known as *Zellballen* pattern which is surrounded by a rich capillary network of reticulin. Electron microscopy reveals the presence of cytoplasmic neurosecretory granules. The neoplastic cells exhibit affinity for chromic acid.^1^ Malignancy is suspected on the basis of distant metastasis as histologically there are no distinct features to differentiate malignant lesions from benign lesions.^3^

On immunohistochemistry, paragangliomas express numerous markers and Chromogranin is ubiquitous in most of the paragangliomas as it is present in the secretory granules. Other markers are Synaptophysin, neuron specific Enolase and S-100 protein, the last one stains the peripheral sustentacular cells.^9^ Chromogranin was negative in our case as extra-adrenal paragangliomas are usually non-functional.

Head and neck paragangliomas can be malignant in 4–19% cases and metastasis occur in 9% cases. Lymphnodes, lungs and bones are the prone sites for metastasis.^10^ Complete surgical excision with disease free margin is the treatment of choice. Arnes et al. showed the recurrence rate to be 10%. Some authors prefer radiotherapy to surgery but it should be reserved as an adjuvant therapy for incompletely excised tumours and patients who are old or unfit for surgeries. Chemotherapy is not effective for this neoplasm and embolisation is used occasionally only to reduce the intra-operative blood loss.^10^ Endonasal endoscopic approach is far superior to traditional approaches like lateral rhinotomy and degloving techniques. It is cosmetically and functionally superior and associated with less morbidities. It also provides a magnified view which helps in better delineation of the area of the tumour attachment and preservation of the important adjacent structures as well. It shortens the hospital stay of the patient and leaves no surgical scar. However, regardless of the surgical approach long term follow-up is essential keeping the aggressive nature of the tumour in mind. In our case the patient was followed up for one year without any endoscopic or radiologic evidence of recurrence.

## Conclusion

We present a case of paraganglioma of nasal septum in a 15 year old boy. Paraganglioma of nasal cavity is an exceptionally rare entity and there have only been three previous reports of this pathology arising from the nasal septum. We conclude that paraganglioma should be included in the differential diagnosis of unilateral vascular nasal mass and nasal septum should be considered as one of the potential yet rare sites for this tumour. Complete surgical excision is not associated with recurrence. Histopathology and immunohistochemistry should be corroborated in doubtful cases.

## Conflicts of interest

The authors declare no conflicts of interest.
